# Activity and Connectivity Differences Underlying Inhibitory Control Across the Adult Life Span

**DOI:** 10.1523/JNEUROSCI.2919-17.2018

**Published:** 2018-09-05

**Authors:** Kamen A. Tsvetanov, Zheng Ye, Laura Hughes, David Samu, Matthias S. Treder, Noham Wolpe, Lorraine K. Tyler, James B. Rowe, for the Cambridge Centre for Ageing and Neuroscience

**Affiliations:** ^1^Centre for Speech, Language and the Brain,; ^2^Cambridge Centre for Ageing and Neuroscience (Cam-CAN), Department of Psychology and MRC Cognition and Brain Sciences Unit, University of Cambridge, Cambridge CB2 3EB, United Kingdom,; ^3^Key Laboratory of Mental Health, Institute of Psychology, Chinese Academy of Sciences, Beijing 100101, People's Republic of China,; ^4^Center for Excellence in Brain Science and Intelligence Technology, Chinese Academy of Sciences, Shanghai 201204, People's Republic of China,; ^5^Department of Clinical Neurosciences, University of Cambridge, Cambridge CB2 2PY, United Kingdom,; ^6^Medical Research Council Cognition and Brain Sciences Unit, Cambridge CB2 7EF, United Kingdom, and; ^7^School of Computer Science and Informatics, Cardiff University, Cardiff CF24 3AA, United Kingdom

**Keywords:** aging, functional magnetic resonance imaging (fMRI), individual differences, inhibitory control, network connectivity, regional activity

## Abstract

Inhibitory control requires precise regulation of activity and connectivity within multiple brain networks. Previous studies have typically evaluated age-related changes in regional activity or changes in interregional interactions. Instead, we test the hypothesis that activity and connectivity make distinct, complementary contributions to performance across the life span and the maintenance of successful inhibitory control systems. A representative sample of healthy human adults in a large, population-based life span cohort performed an integrated Stop-Signal (SS)/No-Go task during functional magnetic resonance imaging (*n* = 119; age range, 18–88 years). Individual differences in inhibitory control were measured in terms of the SS reaction time (SSRT), using the blocked integration method. Linear models and independent components analysis revealed that individual differences in SSRT correlated with both activity and connectivity in a distributed inhibition network, comprising prefrontal, premotor, and motor regions. Importantly, this pattern was moderated by age, such that the association between inhibitory control and connectivity, but not activity, differed with age. Multivariate statistics and out-of-sample validation tests of multifactorial functional organization identified differential roles of activity and connectivity in determining an individual's SSRT across the life span. We propose that age-related differences in adaptive cognitive control are best characterized by the joint consideration of multifocal activity and connectivity within distributed brain networks. These insights may facilitate the development of new strategies to support cognitive ability in old age.

**SIGNIFICANCE STATEMENT** The preservation of cognitive and motor control is crucial for maintaining well being across the life span. We show that such control is determined by both activity and connectivity within distributed brain networks. In a large, population-based cohort, we used a novel whole-brain multivariate approach to estimate the functional components of inhibitory control, in terms of their activity and connectivity. Both activity and connectivity in the inhibition network changed with age. But only the association between performance and connectivity, not activity, differed with age. The results suggest that adaptive control is best characterized by the joint consideration of multifocal activity and connectivity. These insights may facilitate the development of new strategies to maintain cognitive ability across the life span in health and disease.

## Introduction

The preservation of cognitive and motor control is crucial for maintaining well being across the life span (Diamond, 2013). Inhibitory control is critically dependent on frontostriatal circuitry (Aron et al., 2014), in terms of both functional segregation and integration, which can be studied using brain activity and connectivity (Razi and Friston 2016). However, the distinction and complementarity between activity (Zhang et al., 2017) and connectivity (Whelan et al., 2012) within inhibition circuits has yet to be clarified, particularly in terms of the potentially differential effects of age on them (Sebastian et al., 2013). We propose that their joint consideration provides a richer repertoire of brain dynamics underlying cognitive processes than separate consideration of activity and connectivity.

We focus on inhibitory control, which is a central executive function and a determinant of individual differences across multiple cognitive tasks (Miyake and Friedman 2012). Experimental paradigms to isolate inhibitory control from other executive functions typically include abrupt moderations of prepotent responses (Miyake et al., 2000), whether one eventually proceeds to the prepotent action, an alternative action, or no action at all. We assess inhibitory control with a Stop-Signal task, in which participants perform a reaction time task but must occasionally cancel an action after it has been initiated. From the perspective of computational models, the task establishes “race” between the following two largely independent processes: “going” and “stopping” (Logan et al., 2014). Late in a trial, they interact such that stopping can interrupt going leading to no response (Boucher et al., 2007) or delay responses sufficiently to allow reevaluation of the optimal decision under uncertainty or risk (Wiecki and Frank 2013).

The efficiency of inhibitory control changes across the life span (Williams et al., 1999). This arises from differential changes in the speed of going and the probability of successful stopping, which can be summarized in the Stop-Signal response time (SSRT; Verbruggen and Logan 2008). Response inhibition is dependent on distinct but interacting neural circuits that include the right inferior frontal gyrus, pre-supplementary motor area (SMA)/dorsal anterior cingulate cortex (dACC), and basal ganglia (Rae et al., 2014, 2015). These regions are important for inhibiting a motor response in human (Swick et al., 2011) and animal models (Eagle et al., 2008). They are sensitive to selective pharmacological interventions (Ye et al., 2014, 2015) and normal aging (Sebastian et al., 2013). Although studies of functional localization (Swick et al., 2011) and focal brain lesions (Eagle et al., 2008) have suggested that these regions form a circuit or network for inhibitory control, they do not distinguish signals arising from local processing (regional activity) and hierarchically distributed processing (within- and between-network connectivity; Razi and Friston 2016). The latter calls for measures of connectivity in the network related to response inhibition.

Previous studies of individual differences in response inhibition have often focused on a limited number of brain regions (Rae et al., 2015) or constrained networks (Cai et al., 2014), or have examined the contributions of activation and connectivity separately (Zhang et al., 2014). Task-based fMRI studies of response inhibition in aging populations have largely reported changes in inhibition-related activity rather than connectivity (Sebastian et al., 2013; Coxon et al., 2016). This leaves unanswered the question of whether changes in activity and/or connectivity provide a better account of individual differences in inhibitory control and resilience across the life span.

We therefore used a whole-brain data-driven approach to identify both activity and connectivity related to inhibitory control across the life span. We had three related aims. First, to characterize the functional components of response inhibition induced by restraint and cancellation of responses during a well established paradigm, the Stop-Signal/No-Go (SNG) task (Ye et al., 2014, 2015). This unbiased, data-driven approach tested whether these components are similar to previously reported data. Second, to test whether context-dependent activity and context-dependent connectivity predict individual differences in inhibitory control, and do so better than context-independent (spontaneous) connectivity. Third, to test the hypothesis that regional activity and context-dependent connectivity are independent determinants of inhibitory control, and that their joint contribution provides a better model for brain dynamics across the life span.

## Materials and Methods

### Participants

A cohort of 119 healthy human adults was uniformly sampled from a large population-based study of the healthy adult life span (*N* = 658; age range, 18–88 years) in the Cambridge Centre for Aging and Neuroscience (for full details including recruitment strategy, see Shafto et al., 2014). Demographic characteristics of the sample are summarized in Table 1. Ethical approval was obtained from the Cambridge 2 Research Ethics Committee, and written informed consent was given by all participants. Exclusion criteria included poor hearing (threshold, 35 dB at 1000 Hz in both ears) and poor vision (below 20/50 on the Snellen test; Snellen, 1862), a history of serious drug abuse as assessed by the Drug Abuse Screening Test (DAST-20; Skinner, 1982), significant psychiatric disorders (e.g., schizophrenia, bipolar disorder, personality disorder), or neurological diseases (e.g., known stroke, epilepsy, traumatic brain injury).

**Table 1.** Participants' demographic information

	Decile							Statistical tests*^a^*	
	1	2	3	4	5	6	7	K2 or *F* test	*p* value
Age range (years)	18–27	28–37	38–47	48–57	58–67	68–77	78–90		
Gender								0.10	0.996
Men	4 (50)	12 (57.1)	9 (47.4)	10 (52.6)	9 (47.4)	8 (50)	8 (47.1)		
Women	4 (50)	9 (42.9)	10 (52.6)	9 (47.4)	10 (52.6)	8 (50)	9 (52.9)		
Handedness*^b^*								1.82	0.102
Mean/SD	59/67	88/36	82/36	92/11	69/58	98/5	89/19		
Range (minimum/maximum)	−100/100	−65/100	−56/100	70/100	−78/100	87/100	25/100		
Mini-Mental State Examination								2.92	0.011
Mean/SD	29.5/0.8	29.7/0.5	29.2/1	29.2/1	29.3/0.9	28.4/1.3	28.8/1.5		
Range (minimum/maximum)	28/30	29/30	26/30	26/30	27/30	26/30	25/30		

Percentage of the age decile is given in parentheses.

*^a^*Statistical test to indicate whether demographics vary between deciles.

*^b^*Higher scores indicate greater right-hand preference.

#### Stimuli, task, and procedure

**Figure 1.**
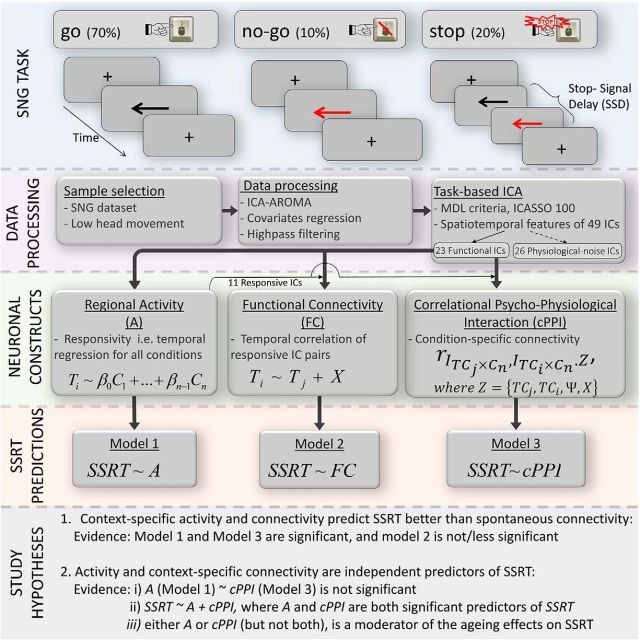
Schematic representation of the task, imaging processing, analysis pipeline and hypotheses in the current study. SSD, Period of variable length to titrate participants' performance at 50% successful response cancellation of all Stop trials (20% of all trials; i.e., the number of successful inhibition trials, unsuccessful inhibition trials, and No-Go trials was similar; ∼10% of all trials, see text); Ti, time course of *i*th component; β, β coefficients; C1, HRF-convolved time course for condition 1; X, regressors for covariates of no interest including head motion, WM, and CSF.

Figure 1 provides a schematic representation of the task and imaging data-processing pipeline. This task assessed cognitive control systems involved in action restraint and action cancellation using No-Go trials (*n* = 40; 10%) and Stop-Signal trials (*n* = 80, 20%, ∼50% of which were successful), respectively, which were randomly interleaved with Go trials (*n* = 360; 70%) during two consecutive scanning runs. On Go participants saw a black arrow (duration, 1000 ms) and indicated its direction by pressing left or right buttons with the index or middle finger of their right hand. On Stop-Signal trials, the black arrow changed color (from black to red) concurrent with a tone, after a short, variable SS delay (SSD). Participants were instructed to withhold button pressing if the arrow was red or became red. The length of the SSD varied between stop trials in steps of 50 ms, and was titrated to participants' performance using an on-line tracking algorithm to maintain a 50% successful response cancellation. In No-Go trials, the arrow was red from the outset (duration, 1000 ms) along with a concurrent auditory tone, equivalent to a Stop-Signal trial with an SSD of 0. The following four key parameters of interest were measured: the rate of Go commission errors (left/right response was incorrect), the mean reaction time of correct Go trials, the rate of No-Go commission errors, and the SSRT.

SSRT was estimated using the block-based integration method to account for skewing and response slowing that may introduce spurious inhibitory differences (Verbruggen et al., 2013). Due to the randomized nature of the task, we redefined blocks to ensure that each block contained 30 Stop-Signal trials. In short, SSRT for each block was estimated by subtracting the mean SSD from the “critical Go RT” and corrected for the Go omission rate (Ye et al., 2015). The critical Go RT was the *n*th RT in the ranked Go RT distribution, where *n* was determined by the mean *p*(respond|signal) and the total number of correct Go trials in each block. We controlled for rare cases where a response in Stop-Signal trials was shorter than SSD (i.e., participants responded before signal for cancellation was given by the arrow color turning from black to red) using the following formula *p*(respond|signal) = (*N*_StopSignal_ − *N*_SuccStop_ − *N*_RT<SSD_)/(*N*_StopSignal_ − *N*_RT<SSD_). Given that a high number of *N*_RT<SSD_ trials would affect the estimation of *p*(respond|signal) and SSRT, we excluded seven individuals having <70 Stop-Signal trials (i.e., >10 *N*_RT<SSD_ trials) in line with the recommended minimum number of Stop-Signal trials required for the block-based integration method (Verbruggen et al., 2013). The number of *N*_RT<SSD_ did not correlate with age (*r* = 0.15, *p* = 0.10).

#### MRI acquisition and preprocessing

Imaging data were acquired using a 3 T Siemens TIM Trio with a 32-channel head coil. A 3D structural MRI was acquired on each participant using a T1-weighted sequence with generalized autocalibrating partially parallel acquisition with the following parameters: acceleration factor, 2; repetition time (TR) = 2250 ms; echo time (TE) = 2.99 ms; inversion time = 900 ms; flip angle α = 9°; field of view (FOV) = 256 × 240 × 192 mm; resolution = 1 mm isotropic); acquisition time, 4 min and 32 s.

For fMRI, echoplanar imaging (EPI) of 314 volumes captured 32 slices (sequential descending order), slice thickness of 3.7 mm with a slice gap of 20% for whole-brain coverage with the following parameters: TR = 2000 ms; TE = 30 ms; flip angle α = 78°; FOV = 192 × 192 mm; resolution = 3 × 3 × 4.44 mm, with a total duration of ∼10.5 min. For preprocessing details, see Taylor et al. (2017). In short, we used Automatic Analysis (AA version 4.0; Cusack et al., 2014) pipelines and modules, which called functions from SPM12 (Wellcome Department of Imaging Neuroscience, London, UK; Friston et al., 2007). The T1 image was initially coregistered to the MNI template, and the T2 image was then coregistered to the T1 image using a rigid-body transformation. The coregistered T1 and T2 images were used in a multichannel segmentation to extract probabilistic maps of six tissue classes: gray matter (GM), white matter (WM), CSF, bone, soft tissue, and residual noise. The native-space GM and WM images were submitted to diffeomorphic registration (DARTEL; Ashburner, 2007) to create group template images. Each template was normalized to the MNI template using a 12-parameter affine transformation. After applying the normalization parameters from the T1 stream to warp preprocessed functional images into MNI space, the normalized images were smoothed using an 8 mm Gaussian kernel (Taylor et al., 2017). EPI data preprocessing included (1) spatial realignment to correct for head movement and movement by distortion interactions, (2) temporal realignment of all slices to the middle slice, and (3) coregistration of the EPI to the participant's T1 anatomical scan (Taylor et al., 2017).

To quantify the total motion for each participant, the root mean square volume-to-volume displacement was computed using the approach of Jenkinson et al. (2002). Participants with ≥3.5 SDs above the group mean motion displacement beyond age effects were excluded from further analysis (*N* = 1). We did not use a strict cutoff for identifying participants with excessive head motion, since (1) framewise measures are dependent on the sampling rate (i.e., repetition time of BOLD data), (2) the degree of motion in our sample during the SNG task is comparable to previous reports (see below), and (3) the use of another conservative approach in the effort to ensure that our findings do not reflect differences in head motion (see below).

In particular, to minimize the impact of head motion on later analysis of connectivity, we took four further processing steps. On a within-subject level, fMRI data were processed using whole-brain independent component analysis (ICA) of single-subject time series denoising, with noise components selected and removed automatically using a priori heuristics using the ICA-based Automatic Removal of Motion Artifacts toolbox (AROMA; Pruim et al., 2015a,b). This was complemented with linear detrending of the fMRI signal, covarying out six realignment parameters, WM and CSF signals, their first derivatives, and quadratic terms (Pruim et al., 2015a). Global WM and CSF signals were estimated for each volume from the mean value of WM and CSF masks derived by thresholding SPM tissue probability maps at 0.75. On a between-subject level, we used group ICA to dissociate task-relevant and other physiological components (see below) and included a subject-specific summary estimate of head movement for each participant (Jenkinson et al., 2002) as a covariate throughout all group-level regression-based analyses (Satterthwaite et al., 2013; Yan et al., 2013; Power et al., 2014; see below).

#### Definition of functional components

We used group ICA to identify components that are activated in a specific experimental conditions and/or contrasts. All participants' data were temporally concatenated and submitted to an ICA analysis with 100 ICASSO (software for investigating the reliability of ICA estimates by clustering and visualization; Himberg and Hyvarinen 2003) iterations, using the Group ICA of fMRI Toolbox (http://mialab.mrn.org/software/gift/index.html; Calhoun et al., 2001). This method decomposes the fMRI signal into a set of independent components, each with a set of subject-specific spatial maps and time courses (TCs), which were standardized using *z*-scoring. The optimal number of components of the ICA decomposition was determined using PCA with minimum description length (MDL) model order selection criteria (Hui et al., 2011). Each spatial map indicates a constellation of voxels (i.e., functional brain regions) that share a common time course over the duration of the experiment, where the strength of the loading value of each voxel reflects the extent to which the given voxel expresses the common time course. The time course of each component was used in a subsequent temporal regression analysis to assess the activity of the component to the cognitive condition of interest, termed here as “component responsivity.”

Physiological noise components were identified and rejected based on spatial and temporal features of the components (Allen et al., 2011, 2014), with reference to the spatial overlap of each component with previously reported network templates (Shirer et al., 2012) and temporal features, using a ratio of 0.9 between high- and low-frequency fluctuations in the signal of the components as a threshold for physiological noise components (Allen et al., 2011, 2014). To further validate our results of the group ICA decomposition in our sample, we split the whole sample to two groups of slow-SSRT and fast-SSRT individuals, according to median SSRT (*N* = 59 in each group). Spatial and temporal features for each subgroup-ICA were compared with those of the main-ICA using the whole sample. Spatial feature similarity was based on the correlation of vectorized spatial maps for each pair of independent components (ICs; e.g., IC_rIFG(Main)_ ∼ IC_rIFG(SlowSSRT)_ and IC_rIFG(Main)_ ∼ IC_rIFG(FastSSRT)_). Next, we tested whether the similarities for each subgroup (with the whole group) differed across subgroups using repeated-measures ANOVA. Temporal feature similarity was based on within-subject and between-subject intraclass correlation coefficients (ICC). Between-subject ICC was based on the (concatenated) time series for a given IC across individuals assigned in the subgroup-ICA and in the whole-group ICA. The within-subject ICC was based on the (vectorized) functional connectivity profile for a given individual in the main-ICA and the corresponding subgroup-ICA. We tested for differences in similarity between slow SSRT and fast SSRT groups using nonparametric one-way ANOVA and repeated-measures ANOVA for between- and within-subject ICCs, respectively. Insignificant results would indicate no differences in the similarity between subgroup-ICAs and the whole-group ICA. All tests for differences in similarity between groups were based on fisher *z*-transformations of the correlation values.

#### Characterizing functional substrates of response inhibition

Two contrasts are commonly used to identify inhibition-related activations, as follows: comparing successful Stop-Signal trials (SuccStop) versus correct Go trials or comparing SuccStop versus unsuccessful Stop-Signal trials (UnsuccStop). Here, we are using the latter approach, as a contrast that has associated differences in neural processes directly with differences in SSRT (Rubia et al., 2003, 2007, Li et al., 2006, 2008; Duann et al., 2009; Boehler et al., 2010; Congdon et al., 2010; Padmala and Pessoa 2010; Whelan et al., 2012). The No-Go trials in the SNG task allow the option for analysis of action restraint, which has been proposed to be anatomically and pharmacologically distinct (Ye et al., 2014, 2015). However, we did not consider the behavioral relevance of activity during No-Go trials, as No-Go performance was near the ceiling across the life span (see Results), while the method we present is suited to examine multivariate influences on individual differences in performance.

Below we describe three types of functional measures for the components of interest (i.e., excluding physiological noise components), as follows: component activity/responsivity, context-independent (spontaneous) functional connectivity, and context-dependent connectivity.

##### Component responsivity.

For each functional component for each participant, we estimated the responsivity index (i.e., the β weight difference between SuccStop and UnsuccStop conditions averaged across the two recording sessions) using a general linear model (GLM; equivalent to voxel-based group-level GLM analysis). Each model included regressors for the onset of the following key parameters: correct Go trials, correct No-Go trials (successfully restrained response), SuccStop (successfully cancelled response), and UnsuccStop (commission error matching arrow orientation). Regressors of no interest included incorrect Go trials (commission and omission errors), incorrect No-Go trials (commission error), early response of SS trials (response shorter than SSD), incorrect SS trials (commission error not matching arrow orientation), six realignment parameters, session index, and standard harmonic regressors that capture low-frequency changes (1/128 Hz) in the signal typically associated with scanner and physiological noise. The events in the trials were modeled using the shape of the hemodynamic response function (HRF) as implemented in SPM12.

To identify group-level functional components of response inhibition, we computed the responsivity index of the contrast SuccStop > UnsuccStop across all 119 individuals (one-sample *t* test, corrected for multiple comparisons using false discovery rate (FDR) correction with an α = 0.01). It should be noted that this contrast identified condition-relevant activity differences between Unsuccessful and Successful Stop-Signal trial types. To further determine age-dependent components, we used a robust linear regression with component responsivity and covariates of no interest (gender, handedness, and level of education) as independent variables, and age as a dependent variable, where FDR correction at α = 0.05 was used to account for multiple comparisons. The significance level in the analysis of group effects (*p* < 0.01, FDR corrected) was more conservative than the analysis of age effects (*p* < 0.05, FDR corrected) given the different nature of the tests (i.e., differences between group means vs correlating a continuous variable), resulting in a high magnitude of group effects relative to age effects. Importantly, the same threshold levels were used for context-dependent and context-independent functional connectivity to ensure comparability in their analysis with SSRT performance (see below).

##### Context-independent functional connectivity.

To derive a subject-specific measure of context-independent functional connectivity between each pair of responsive components (e.g., between time courses of TC*_i_* and TC*_j_* of the *i*th and *j*th ICs, similar to resting-state functional connectivity), we used multiple linear regression (MLR) with TC*_j_*, regressors for all experimental conditions of interest and no interest (see Component responsivity), six realignment parameters, WM, CSF, and session run as independent variables, and TC*_i_* as a dependent variable for each participant (for more details, see Fornito et al., 2012). Group and age effects of each connection pair were identified in a manner similar to that for the responsive components (see Definition of functional components), where FDR correction at α levels 0.01 and 0.05 were used to account for multiple comparisons, respectively.

##### Context-dependent functional connectivity.

Context-specific functional connectivity analyses have typically used correlational psychophysiological interactions (cPPIs; Fornito et al., 2012). cPPI analysis provides confirmatory evidence for the pattern of connectivity modulation between IC pairs responsive in the contrast SuccStop > UnsuccStop. These cPPI measures are based on the partial association between their interaction terms (*I*_TCj×Cn_ = TC*_i_*×Contrast; *I*_TC_*_i_*_×Cn_ = TC*_i_* × Contrast), after being orthogonalized against experimental design (Ψ), IC time series (TC*_j_*; TC*_i_*) and other covariates (*C*, including head motion, WM, and CSF signal; i.e., *r*_*I*_*TC_j_*×*C_n_*_, *I*_*TC_i_*×*C_n_*_·*Z*_, where *Z* = {TC*_j_*, TC*_i_*, Ψ, *C*} and the period in the subscript separating the correlated variables and the controlled for variable; Fig. 1; Fornito et al., 2012). Consistent effects across subjects were then tested using two-sample *t* tests in a model that assumed neither independence nor equal variance between the conditions.

We assessed whether context-specific connectivity between each pair of responsive ICs (1) were expressed consistently across all 119 individuals, using a one-sample *t* test on the group level; and (2) changed as a function of age, using multiple linear regression with age as a dependent variable and cPPI between IC pairs as an independent variable (similar to the analysis of responsive components (see Definition of functional components and Context-independent functional connectivity). Connections at significance levels of *p* < 0.01 and *p* < 0.05 (FDR corrected) were considered for subsequent group effects and age effects analysis, respectively.

#### Behavioral relevance of functional substrates of response inhibition

The predictive performance of these three sets of functional measures (see Characterizing functional substrates of response inhibition) on individual variability in SSRT (see Stimuli, task, and procedure) was assessed separately for component responsivity (Model 1), spontaneous connectivity (Model 2), and context-specific connectivity (Model 3), and was compared across models. For the significant models, we then constructed a set of mixture models *post hoc* to investigate their joint predictive contribution; that is, given that component activity and cPPI predict performance reliably (Model 1 and Model 3 were significant), a new model will test how well activity and cPPI can jointly predict performance (Model 4). For this purpose, we used MLR with well conditioned shrinkage regularization (Ledoit and Wolf 2004; Blankertz et al., 2011) and permutation-based 10-fold cross-validation (Lemm et al., 2011) to investigate the corresponding structure coefficients (Thompson and Borrello, 1985) of activity and connectivity to performance across the life span. We used a dataset-wise permutation-based cross-validation scheme (Etzel and Braver, 2013) to enable comparison between models with varying complexity (Browne, 2000; Zucchini, 2000). Specifically, we adopted a three-stage procedure, where in the first stage we used MLR in the training set (9 of 10 samples) to produce parameter weights defining a latent variable of the functional measures that highly correlates with SSRT. These parameter weights from the training data were then used in a second stage to estimate the subject scores for subjects left out of the training set (i.e., testing sample, the 10th sample). We repeated the first two stages for each testing sample, so that we estimate subject scores for each subject (equivalent to predicted value *ŷ* in regression). In the final stage, we correlated subject scores to SSRT (predicted value *ŷ* and observed value *y* in the regression, respectively). Therefore, the reported *r* values for various models are always based on a bivariate correlation between observed and predicted SSRTs (i.e., same model complexity regardless of the initial number of predictors in the model; overfitting in more complex models may only occur in the first stage, not in the third stage, of this approach; Zucchini 2000; for more details, see Browne 2000). In addition, the model significance was evaluated against its null distribution based on 1000 permutations of the SSRT values following the three-stage procedure.

To further minimize the non-negligible variance of k-fold cross-validations and to enable statistical comparisons between various models, we repeated each k-fold (and its 1000 permutations) 1000 times with random partitioning of the folds to produce an *R* value distribution for each model. Model comparison was based on the mean difference model evidence for correctly labeled SSRTs, compared with the null distribution of mean differences across permuted SSRTs (Nichols and Holmes 2002).

To further investigate whether the reliable associations between functional measures and behavioral performance were age dependent and/or age independent in nature (i.e., over and above the effects of aging), we used a moderation analysis. Specifically, we constructed a multiple linear model where subject scores (the linear combination of functional variables correlated with behavioral performance for a given model), age, and their interaction (subject scores × age) were used as independent variables and behavioral performance (i.e., SSRT) was used as a dependent variable. Covariates of no interest in the MLR and moderation analysis included gender, handedness, level of education, mean head movement, and mean response time on Go trials.

## Results

### Behavioral data

**Figure 2.**
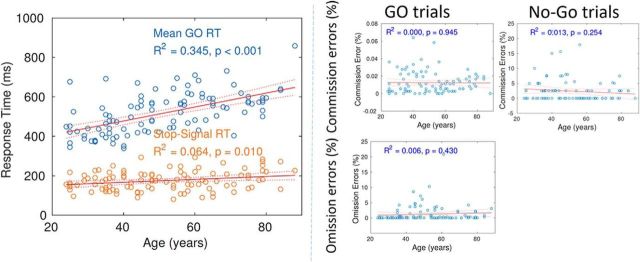
Latency (left) and accuracy (right) measures for Go, No-Go, and Stop-Signal trials of each subject, where each subject's performance is denoted with a circle in the scatter plots and red solid lines denote linear trends across the lifespan with corresponding effect sizes; denoted lines are 95% confidence bounds for the fitted coefficients.

In accordance with previous studies, there were strong age-related differences in GoRT and SSRT but not in Go or No-Go accuracy (Fig. 2). The average SSRT and *p*(respond|signal) were 171 ms and 47%, respectively, both of which were within the range of the values reported in previous studies of healthy adults (Whelan et al., 2012; Hu et al., 2014; Zhang et al., 2015). The results indicated that life span trends for the inhibition of a speeded response are independent from those governing its execution, corroborating previous findings (Williams et al., 1999).

### Head motion during SNG task

Head motion during the SNG task correlated positively with participants' age (*r* = 0.50, *p* < 0.001), and was not statistically different from their head motion during resting state and the Cattell fMRI acquisitions (Geerligs et al., 2017; Samu et al., 2017), as informed by repeated-measures ANOVA (*F* = 1.11, *p* = 0.332). Head motion was significantly lower during movie watching (Campbell et al., 2015) relative to resting state, SNG, and Cattell tasks (*F* = 14.44, *p* < 0.001), which is consistent with previous reports showing that individuals are more compliant during movie watching (Vanderwal et al., 2015). Moreover, head motion across the three cognitive states (resting state, movie watching, and Cattell task) was highly correlated with head motion during SNG (*r* = 0.81, *p* < 0.001) and did not interact with age (*F* = 1.84, *p* = 0.139). The results indicate that head motion in our sample during SNG is comparable to the head motion during other cognitive states (Campbell et al., 2015; Geerligs et al., 2017; Samu et al., 2017).

### Brain components responsive to response inhibition

The optimal number of components (*n* = 49) was detected with MDL criteria, supported by high-stability indices across 100 ICASSO runs (mean = 0.97, SD = 0.02). To further validate that the group ICA decomposition was not biased by the overall sample, we explored the similarity of spatial and temporal features from the main-ICA with two subgroup-ICAs, based on a median split of SSRT (see Materials and Methods). On average, the correlation in spatial overlap across responsive ICs (see below) between subgroup-ICA and the main-ICA was very high (mean *r* values of 0.975 and 0.972 for fast-SSRT and slow-SSRT subgroups, respectively) and the similar pattern across ICs between subgroups did not differ (*F* = 0.08, *p* = 0.775). The between-subject ICC indicated high levels of temporal similarity with the main-ICA across responsive ICs (mean *r* = 0.904 and 0.893 for fast- and slow-SSRT subgroups, respectively) and showed no significant difference between subgroups (*F* = 1.16 and *p* = 0.287). Finally, the within-subject ICC indicated a high correspondence of the functional connectivity pattern for individuals in subgroup-ICA and main-ICA (mean *r* value 0.906 and 0.895 for fast- and slow-SSRT subgroups, respectively), which did not differ between subgroups (*F* = 1.58, *p* = 0.118). The above findings indicate the high stability of spatial and temporal decomposition of the data based on group ICA across all individuals in our sample. The exclusion of physiological noise components (see Materials and Methods) left 23 functional components. After fitting the task events to the context-dependent (task-specific) time course of each component, we calculated a responsivity index (i.e., the β weight difference between conditions of interest) for each functional component.

**Figure 3.**
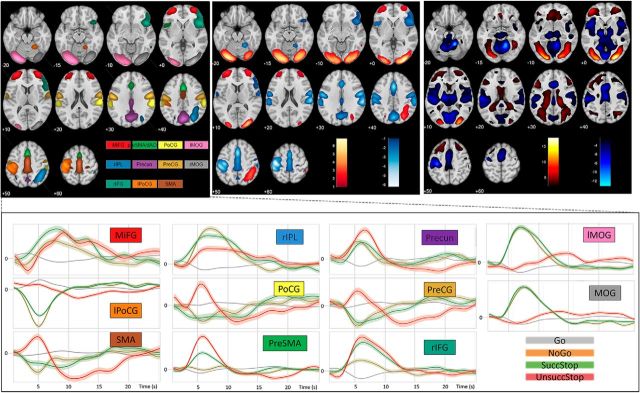
Top left, Group-level component responsivity (difference in condition-specific component activity across contrast) for the contrast SuccStop > UnsuccStop, where each component is shown in a unique color scheme. Lighter color within a given color scheme reflects a higher loading value for a given voxel (i.e., a stronger association between the time course of the voxel and the time course of the component). Top center, Direction of reactivity of the reactive components—higher (hot colors) and lower (cold colors) reactivity for SuccStop > UnsuccStop, where the reactivity of the component is weighted by the loading value of the voxel on the component (see Materials and Methods). Top right, Group effects using traditional univariate GLM analysis in SPM. Regional brain activation for SuccStop > UnsuccStop, warm color scheme; and for UnsuccStop > SuccStop, cold color scheme. Thresholded at a significance level of *p* < 0.001, uncorrected. There was a high spatial overlap in gray matter activations between the group ICA and GLM methods (*r* = 0.59, *p* < 0.001). The differences between the two approaches originated mainly in white matter, vascular, and CSF territories, indicating that group ICA may be less sensitive to individual and age-related differences of physiological signals of non-neuronal origin than traditional univariate GLM analysis. Bottom, Event-related time courses for four types of trials.

**Figure 4.**
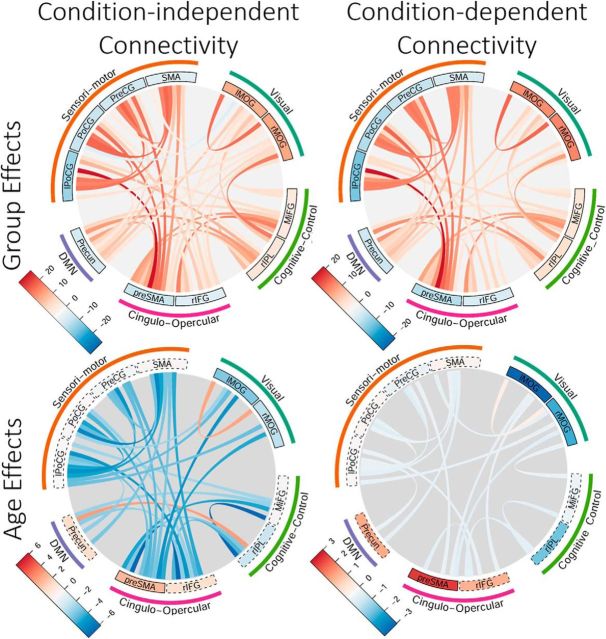
Chord diagrams representing group and age effects of spontaneous connectivity (left) and context-specific connectivity (SuccStop > UnsuccStop; right) for responsive components. Component responsivity (difference in context-dependent component activity across contrasts) for the contrast SuccStop > UnsuccStop is shown in the inner band (higher and lower responsivity are shown in hot and cold colors, respectively), with components having significant group (main) effects (top) and age effects (bottom) denoted with a black outline (*p* < 0.01, FDR corrected). For labels of components (inner band) and their network correspondence (outer band), see text. l, Left; r, right.

Brain components reactive to SuccStop > UnsuccStop contrast across participants are shown in Figure 3. We assigned a heuristic descriptive name to each of these components, based on its highest correspondence with previously reported functional region-specific templates based on task-based fMRI data (Shirer et al., 2012). Most of the components were restricted within a single template region (i.e., node within a network), suggesting that the resulting components reflect the activity from an individual functional region, rather than synchronized network activity. Components consistently responsive to the SuccStop > UnsuccStop contrast across individual participants included the following: (1) a set of regions showing higher activity during UnsuccStop: right inferior-frontal gyrus (rIFG), pre-SMA/dACC, and sensorimotor regions [left posterior central gyrus (lPoCG), right PoCG, bilateral precentral gyrus (Pre-CG), and SMA], and precuneus (Precun); and (2) a set of regions showing higher activity during SuccStop: right intraparietal lobule, bilateral inferio-medial frontal gyrus (MiFG) and bilateral medial occipital gyrus (MOG; Fig. 3, the spatial extent of the components and their event-related time courses for different conditions). The ICA results were highly consistent with the results using the traditional univariate GLM approach in SPM12 (spatial overlap with *r* = 0.59, *p* < 0.001; Fig. 3, top right). In terms of aging, we found that the task-positive components (functional components positively associated with task performance) were activated to a lesser extent by older adults, accompanied by a weaker deactivation (or suppression) of task-negative components. However, only one component survived correction for multiple comparisons, namely the pre-SMA/dACC, with an age-related decrease in absolute responsivity values, between SuccStop and UnsuccStop (Fig. 4, bottom left).

### Context-independent functional connectivity of responsive components

We next looked at context-independent (also called spontaneous) functional connectivity over the duration of the experiment, while controlling for variability associated with the experimental design and events of no interest (see Materials and Methods). This analysis revealed that the SNG task positively modulates context-independent functional connectivity between components responsive to the SuccStop > UnsuccStop contrast (Fig. 4, top left). In particular, we observed strong spontaneous connectivity between ICs within each network (high within-network connectivity) and spontaneous connectivity between some ICs belonging to different networks (between-network connectivity). This included modulated spontaneous connectivity of (1) sensorimotor network with cingulo-opercular network ICs and (2) cognitive-control ICs with cingulo-opercular network, default mode network, and visual network. There was a weak spontaneous connectivity between cognitive control and sensorimotor ICs and between primary sensory network connections (sensorimotor vs visual networks components). In terms of aging, spontaneous connectivity between all but two ICs decreased as a function of age (Fig. 4, bottom left).

### Context-dependent functional connectivity of responsive components

To address the extent to which activity (i.e., component responsivity) and connectivity of the 11 responsive components are important for successful performance, we estimated context-specific connectivity (i.e., cPPI) between each pair of responsive components (see Materials and Methods). For each pair of responsive components, we estimated context-dependent connectivity by contrasting connectivity during successful and unsuccessful stopping (SuccStop > UnsuccStop), indicating the changes in connectivity strength between unsuccessful and successful stopping, using a significance level of *p* < 0.05 (FDR corrected). Context-dependent connectivity between pairs of responsive components was stronger during SuccStop compared with UnsuccStop events with a pattern of connectivity modulation very similar to the pattern of context-independent connectivity (Fig. 4, top right; see Context-independent functional connectivity). Even though there was a significant decrease in the context-dependent connectivity differences between SuccStop and UnsuccStop, the effects of aging on context-specific connectivity were weaker than those on context-independent spontaneous connectivity (in terms of effect sizes and number of connections; Fig. 4, bottom right), indicating that the ability to efficiently modulate connectivity between conditions is fairly preserved across the life span.

### Individual variability of response inhibition determined by brain function

#### Component responsivity

**Figure 5.**
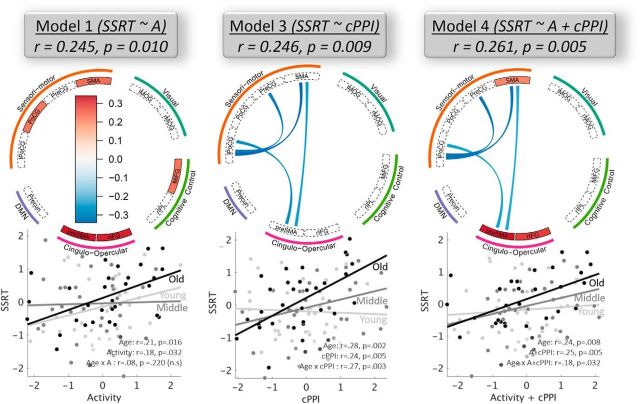
Significant models of brain measures predicting individual variability in response inhibition (SSRT). Specifically, the significant models include responsivity only (A in Model 1, larger activity in CO and SM nodes during UnsuccStop vs SuccStop was associated with faster SSRT), context-specific connectivity (cPPI in Model 3, larger connectivity modulations between SuccStop and UnsuccStop were associates with faster SSRT), and their joint contribution (Model 4). While context-specific connectivity predicted individual, age-related, and age-moderated variances in SSRT, activity was a significant predictor of the first two only. Below each circular plot, a scatter plot of corresponding bivariate correlation for three equally sized age groups is shown. The relationship between SSRT and connectivity is higher for older (formally confirmed by moderation analysis, see Age × cPPI in Model 3), suggesting that good performance in older adults relies more strongly on a good connectivity profile between functional components. The model with functional connectivity (Model 2) is not shown as it was not significant. l, Left; r, right.

**Figure 6.**
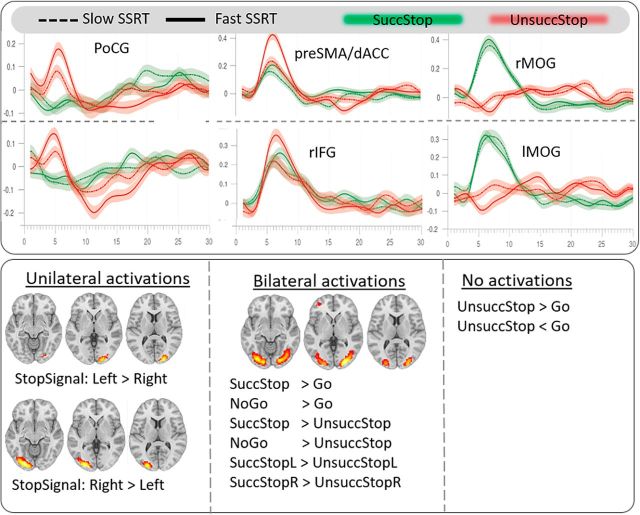
Top, Event-related time courses for six components during SuccStop (green lines) and UnsuccStop (red lines) across two SSRT groups of individuals (continuous line, fast SSRT; dotted line, slow SSRT). Bottom left, Additional set of analyses demonstrating the activity exclusively in bilateral MOG, as observed during SuccStop > UnsuccStop (Figs. 3, 4). Left, Unilateral activity in medial occipital areas increases in response to arrow orientation to the contralateral side, indicating that the involvement of MOG might be implicated in stimulus response. Right, No activation differences in MOG due to perceptual processing in stimulus differences (e.g., red vs black arrow, UnsuccStop > Go or Go > UnsuccStop). Center, Bilateral activations during correctly performed trials vs error/baseline trials, independent of stimulus lateralization (i.e., SuccStop Left > UnsuccStop Left). Right bottom, Additional set of analyses demonstrating the behavioral relevance of regional activity during SuccStop > Go in terms of predicting individual variability in response inhibition, SSRT (identical to Model 1 in Fig. 5). The correlation between regional activity of the 19 ICs responsive to SuccStop > Go contrast and SSRT was significant (*r* = −0.270, *p* = 0.005), of which only 4 ICs contributed significantly. Namely, higher activation in right MiFG and left MOG during SuccStop vs Go trials was associated with faster SSRTs. In addition, lower activation in left PoCG and Pre-CG during SuccStop vs Go was associated with faster SSRTs. Moderation analysis revealed that the association between regional activity and performance remained (*r* = 0.117, *p* < 0.001) beyond the effects of aging (*r* = 0.144, *p* < 0.001), which did not vary across the life span (i.e., insignificant moderation effect, *p* = 0.24). Furthermore, the test results using context-dependent connectivity among all 19 ICs to predict SSRTs in multiple linear regression (equivalent to Model 3 in Fig. 5) was insignificant, indicating that the contrast SuccStop vs Go trials might be a less sensitive brain-wide connectivity modulation with behavioral relevance to inhibition control.

The behavioral relevance of all responsive components (*n* = 11) was assessed by examining their relationship to the SSRT. We used an MLR (see Materials and Methods) with 10-fold cross-validation to determine the joint contribution of shared variance (i.e., structure coefficients) in activity across all responsive components to SSRT variability across all participants. Differences in activity for the contrast SuccStop > UnsuccStop were relevant to SSRT for 7 of 11 responsive components (*r* = 0.245, *p* = 0.010; Fig. 5, Model 1). Moreover, the nature of this relationship remained significant (SSRT approximate subject scores of activity in Model 1: *r* = 0.18, *p* = 0.032) over and above the effects of age and other covariates (handedness, gender, head movement, and response times on correct Go trials), indicating that the identified set of functional components are fairly robust to determine individual variability in SSRT across the life span. The interaction term between age and subject scores of activity was not significant (*r* = 0.08, *p* = 0.220; Fig. 5, Model 1), suggesting that the collective regional activity of those components does not change its importance to perform the SNG task across the life span. The components explaining unique variance of SSRT included PoCG, SMA, rIFG, pre-SMA/dACC, and MiFG. In particular, the difference in activity during UnsuccStop, but not during SuccStop, was associated with SSRTs (see time courses for groups of fast and slow SSRT; Fig. 6 top), indicating that individuals with higher activity during UnsuccStop (relative to SuccStop) had faster SSRTs. This suggests that the recruitment of these components during UnsuccStop trials is an important determinant of SSRT.

#### Context-independent functional connectivity

To test whether context-independent functional connectivity between responsive components determines individual differences in inhibitory response, we used multiple linear regression with SSRT as a dependent variable (Model 2). Context-independent functional connectivity between the responsive components showing significant group and age effects was the independent variable (*n* = 47; Fig. 4, right, significant connections). Covariates of no interest included handedness, gender, head movement, and response times on correct Go trials. Model 2 was not significant (*r* = 0.09, *p* = 0.261), suggesting that, unlike regional activity, context-independent functional connectivity does not account for individual variability in the SSRT.

#### Context-dependent functional connectivity

Finally, we tested whether context-specific functional connectivity between responsive components reliably predicted variability in the SSRT across the life span (Model 3). We used SSRT as a dependent variable and cPPI between the responsive components with significant group and age effects as independent variables (*n* = 41; Fig. 4, right, significant connections). Covariates of no interest included handedness, gender, head movement, and response times on correct Go trials. Model 3 was significant (*r* = 0.246, *p* = 0.009; Fig. 5, Model 3), suggesting that context-specific connectivity is a determinant of SSRT. Differences in connectivity between SuccStop and UnsuccStop were observed in the following pairs of components: lPoCG–Pre-CG; lPoCG–SMA; pre-SMA/dACC–SMA; and lPoCG–pre-SMA/dACC. In particular, all connections showed an increased difference in connectivity between SuccStop and UnsuccStop with faster SSRTs (note that these components showed stronger connectivity during SuccStop versus UnsuccStop; Fig. 4). In other words, individuals with weak connectivity modulation within the somatomotor network between SuccStop and UnsuccStop required a longer time to inhibit a prepotent motor action.

To further investigate the nature of the association between SSRT and context-dependent connectivity, we conducted a *post hoc* moderation analysis including age, subject scores of cPPI from Model 3, and their interaction (age × cPPI subject scores) as independent variables, and SSRT as the dependent variable. Variables of no interest in the model included handedness, level of education, gender, and mean head displacement. The results are shown in Figure 5 (Model 3, Moderation). Subject scores of cPPI were significantly associated with SSRT after accounting for age and other covariates (*r* = 0.24, *p* = 0.005), suggesting that context-specific connectivity, together with regional activity (see Component responsivity) contribute to individual differences in SSRTs across the life span (i.e., over and above the effects of aging). Interestingly, the interaction term between age and cPPI subject scores predicted unique variance in SSRT variability (age × cPPI: *r* = 0.27, *p* = 0.003). In other words, increasing age strengthened the relationship between SSRT and context-specific connectivity.

In summary, while both regional activity and context-specific connectivity were associated with individual and age-related differences in SSRT, only context-specific connectivity interacted with age as a determinant of performance. Context-specific connectivity therefore provided additional information to that of regional activity in terms of explaining variability in SSRTs over the life span. This finding also indicates that regional activity and connectivity may predict independent effects on the SSRT, which we formally tested in the following section.

#### Joint contribution of activity and context-dependent functional connectivity

To investigate whether regional activity and context-specific functional connectivity provide unique determinants of SSRT variability, we constructed an additional model, which was similar to Models 1, 2, and 3, except that the set of SSRT predictors included both regional activity and context-specific functional connectivity (Model 4). Model 4 was significant (*r* = 0.261, *p* = 0.005; Fig. 5, Model 4), suggesting that context-specific activity and connectivity are determinants of SSRT. The number of independent variables in Model 4 was higher than in Model 1 and Model 3 (*n* = 52, 11 components for responsivity and 41 connections for connectivity, which were significantly different between SuccStop and UnsuccStop; Fig. 4, top right). If activity and connectivity predicted the same source of variance in the SSRT, this would lead to reduced performance of Model 4 relative to the simpler models, Model 1 or Model 3 (since the 10-fold cross-validation method controls for overfitting; see Materials and Methods). The results showed that Model 4 performed better than both Model 1 and Model 3, with average *r* values across 1000 CV partitions of *r* = 0.255, *r* = 0.224, and *r* = 0.288, respectively, for Model 1, Model 3 and Model 4. The difference in *r* value distributions was statistically significant between Model 4 and Model 1 (*t* = −8.30, *p* = 0.002) and Model 4 and Model 3 (*t* = −13.91, *p* < 0.001) compared with their mean difference null distributions, indicating that the joint prediction of activity and connectivity explains a significantly larger portion of SSRT variance compared with their predictive values alone.

In support of the above, we tested for an association between activity and connectivity (i.e., whether there is a relationship between the subject scores of activity in Model 1 and those of context-specific connectivity in Model 3). A significant correlation between the two types of subject scores would indicate that activity and connectivity may explain the shared variance in SSRT. However, there was no relationship between the two sets of subjects scores (*r* = 0.01, *p* = 0.951), confirming that activity and connectivity are independent predictors of SSRT. The activity in rIFG and pre-SMA/dACC together with connectivity among lPoCG-PreCG, lPoCG-SMA, and SMA–pre-SMA/dACC were significant predictors of individual and age-related differences in SSRT (Fig. 5, Model 4). Furthermore, the interaction of brain scores with age was significantly associated with SSRT (*r* = 0.18, *p* = 0.032). In other words, the relationships among SSRT, context-dependent connectivity, and regional activity were strengthened in older adults.

## Discussion

The success of cognitive and motor control requires the engagement of a diffuse network comprising prefrontal, premotor, and motor regions. The principal and novel results of this study are that (1) individual differences in inhibition performance correlated with both the degree of activation of these regions, and the degree of connectivity between them; and (2) the effects of age on inhibition performance were determined by the activity of the inferior frontal gyrus and dorsomedial prefrontal cortex, and the modulation of connectivity between these and sensorimotor regions. While task-related differences in context-dependent and context-independent connectivity correlated across the group, the context-dependent connectivity was less influenced by age overall. However, the age-related variance in context-dependent connectivity was a progressively more important determinant of performance differences in older adults. These results are based on a population-based cross-sectional cohort, and cannot directly speak to individual subjects' progression over time. The following discussion of age effects is therefore restricted to the effects of age and its correlates, as assessed across individuals, rather than the dynamic process of individual aging per se.

### Differences in activity of inhibition control regions predict performance

Task-evoked responses in 11 regions differentiated successful from unsuccessful Stop-Signal trials. Right IFG and pre-SMA/dACC—commonly associated with response inhibition—were activated in both conditions, but showed reliably elevated activation for unsuccessful versus successful inhibition (Congdon et al., 2010; Erika-Florence et al., 2014) with behavioral relevance (Li et al., 2008; Duann et al., 2009). These results persisted after accounting for the effects of age and other covariates that might introduce artificial associations between brain and behavioral measures, and corroborate previous group comparisons of fast and slow SSRTs (Congdon et al., 2010). We found no evidence for age-dependent variation in the brain activity–behavior relationship, suggesting that while regional activity in cognitive control regions declines with age (Sebastian et al., 2013), it may not change its importance for performance across the life span.

Premotor and sensorimotor regions also showed elevated activations for unsuccessful versus successful stopping (Congdon et al., 2010). Given that there was no difference in activity between conditions involving motor response (e.g., UnsuccStop vs Go) and between conditions involving no motor response (e.g., SuccStop vs No-Go; data not shown, but available on request), these findings likely reflect the differences in activity between trials with motor response versus trials with no motor response. An additional set of regions with enhanced activity during SuccStop versus UnsuccStop, including the right inferior parietal lobule, bilateral middle occipital gyrus, and bilateral middle frontal gyrus (Fig. 3), showed sensitivity to the orientation of the arrow stimulus in our experiment (Fig. 6, bottom left). Further, these were consistent across all correctly performed trials (irrelevant of the condition) versus all error type and baseline trials, which may reflect attentional readiness or trial-to-trial variability, rather than response inhibition (Jans et al., 2010).

### Differences in context-dependent connectivity relate to performance

The change in connectivity related to inhibition was greater for SuccStop versus UnsuccStop, which is suggestive of relative network segregation during UnsuccStop (weaker connectivity with higher activity; Toni et al., 2002) and more integration during SuccStop. We propose that flexible integration between functional components is important for the performance of executive functions, including but not limited to inhibition (Rowe et al., 2007). Importantly, the differences in the strength of connectivity between prefrontal and premotor/motor regions predicted performance. Smaller differences of connectivity between SuccStop and UnsuccStop led to a longer SSRT (i.e., individuals with weaker connectivity modulation were less able to stop a response after it had been initiated). Our brain-wide approach suggests that the connectivity between widespread regions, not only regions with extreme variability in response inhibition or marked lesion effects (Duann et al., 2009; Congdon et al., 2010; Rae et al., 2015), is an important factor in performance. Activity and connectivity in these regions are indicative of subtle differences in response inhibition, including life span variability in inhibitory performance.

Older adults were less efficient at stopping their actions in terms of SSRT, although the aging effects on Go reaction times were proportionally larger and individual differences are marked (Fig. 2). We propose that this effect of age is due to less dynamic modulation of connectivity between conditions, which is in agreement with emerging evidence of decreased segregation of networks across the healthy adult life span (Chan et al., 2014; Geerligs et al., 2015; Tsvetanov et al., 2016). Our data indicate less brain segregation across the life span in both context-dependent and context-independent connectivity. Furthermore, our findings suggest that having the ability to modulate connectivity between different brain states/conditions facilitates performance, extending the idea about variability in neural processing based on signal variability of neural BOLD activity (Grady and Garrett, 2014) to connectivity.

We speculate that the connectivity modulation between both conditions may arise during the SSRT period, reflecting efficiency in inhibitory processing, where strong connectivity leads to successful inhibition (i.e., SuccStop), while weaker connectivity leads to irrevocable commitment to a highly activated and prepotent response (i.e., UnsuccStop). Alternatively, the difference in connectivity strength associated with SSRT may arise later, from another process that influences poor response inhibition (Congdon et al., 2010); a smaller difference between SuccStop and UnsuccStop (i.e., hyperconnectivity) may reflect an ongoing inhibitory information transmission between pre-SMA and motor regions after Stop-Signal, which would otherwise be suppressed through efficient disinhibition of the (inhibitory) projections to motor regions. In terms of aging, such a pattern of overcoupling may reflect the engagement of a network to support processes irrelevant to the task at hand. For example, when needing to suppress salient distractors, older adults show deficits (Tsvetanov et al., 2013) that may reflect inefficient inhibition of early visual cortices from intraparietal regions leading to attentional capture (Madden et al., 2014).

Both regional activity and context-dependent connectivity were linked to interindividual and age-related variability in performance. A moderation analysis indicated the importance of the interaction between age and connectivity on performance (over and above the effects of individual and age-related differences). Improved stopping efficiency in older adults relied more strongly on the modulation of connectivity within the motor and cognitive control networks. Indeed, the measures of connectivity may be more relevant than regional activity for predicting the preservation of performance across the life span (Tsvetanov et al., 2016; Samu et al., 2017). Collectively, the separate consideration of activity and connectivity provides only a limited repertoire of brain dynamics and their resilience across the life span, to which we turn next.

### Complementary effects of activity and connectivity on performance

We tested the joint contribution of inhibition-related activity and connectivity. Both regional activity in prefrontal regions and connectivity among motor, premotor, and prefrontal cortices were associated with behavioral variability (Model 4). This is consistent with the hypothesis that inhibition-related activity and inhibition-related connectivity are independent determinants of performance across the life span. This was corroborated by our individual analysis of both brain measures (considered separately, Model 1 and Model 3), that (1) there was no association between the subject scores of activity and connectivity, and (2) task-related differences in connectivity (Model 3), but not in regional activity (Model 1), moderated the effects of aging on cognitive performance. Therefore, the joint consideration of regional activity and connectivity provides insights over and above their individual consideration, including the modulators of behaviorally relevant neural systems across the life span.

In line with this, we propose that age-related change in cognitive control is best characterized by the joint analysis of multifocal activity and connectivity within distributed brain networks. This view also predicts that there may be differential patterns at either the regional or inter-regional level. Each one is helpful to understand a specific process, differences across the life span, or disease stages. Furthermore, approaches capable of characterizing the joint contribution of regional activity and connectivity of fMRI signals may provide a principled means to characterize dynamic network communication, and how they may relate to existing electrophysiological phenomena (Voytek and Knight 2015).

### Further considerations

We demonstrated that ICA and univariate GLM analyses of regional activity were highly consistent (Fig. 3; Whelan et al., 2012; Zhang et al., 2015). However, the ICA approach has potential advantages over a GLM approach. For example, the linear models identified a large portion of voxels that are unlikely to indicate neuronal responses (e.g., vascular and/or CSF) being significantly modulated by experimental conditions. The ICA method is less vulnerable to fluctuations in physiological signals (Fig. 3), which may otherwise confound fMRI studies of aging (Tsvetanov et al., 2015, 2016; Geerligs and Tsvetanov 2016; Geerligs et al., 2017).

We found no evidence for the association between context-independent connectivity and performance, in contrast to previous studies (Kelly et al., 2008; Fornito et al., 2012; Tsvetanov et al., 2016). This may reflect our focus on responsive components in the SNG task. Spontaneous connectivity may thus remain an important determinant of other cognitive functions and may need to be considered jointly with context-dependent connectivity to fully characterize neurocognitive preservation across the life span (Geerligs and Tsvetanov 2016).

### Concluding remarks

We show that behaviorally relevant and age-dependent individual differences in response inhibition can be better predicted by the joint consideration of activity and connectivity in distributed brain networks. The state of activity and connectivity in these networks is critical for dissociating multiple sources of individual differences in response to inhibition. Importantly, connectivity was a sole predictor of age-moderated variability in stopping ability, indicating that improved stopping efficiency in older adults relied more strongly on the modulation of the connectivity of motor and cognitive control networks. The joint consideration of activity and connectivity within distributed networks provided a richer repertoire of brain dynamics across the life span with implications for understanding the normal process of aging and potentially for neurodegenerative disorders.
